# Foreword

**DOI:** 10.3109/03009731003672581

**Published:** 2010-03-10

**Authors:** Oreland Lars

**Affiliations:** Prof. emeritus, Guest Editor Past Head of Department of Pharmacology and of Department of Neuroscience

In 1997, it was decided at the Medical Faculty of Uppsala University that the number of departments should be reduced from over forty to about ten. One rationale for this decision was that some departments were too small to be able to carry the responsibilities with regard to economy, administration and long-term care of employees. Another rationale was the hope that larger departments would facilitate contacts between different research groups with beneficial effects on research. Since there were several departments with a strong inclination towards research within the field of neuroscience, eleven previously independent departments were merged into one Department of Neuroscience. This new department had the unusual feature of being a mixture of former clinical departments, such as Psychiatry, Neurology, Ophthalmology etc. with former preclinical departments such as Pharmacology, Medical Developmental Biology, part of Physiology etc. The reorganization took place in 1998 and this issue of the Uppsala Journal of Medical Science (UJMS) has been created in order to celebrate the recent ten-year jubilee. It is hoped that this issue of UJMS will demonstrate the wide-spread research areas within the department, as well as the fact that UJMS is open to publications of clinical, as well as preclinical papers within all areas of medicine.

