# Supplementary material for: Foreword
Source: Ups J Med Sci. 2010 Mar 10;115(1):1. doi: 10.3109/03009731003672581 (PMC2853348; doi:10.3109/03009731003672581)
Supplement: Related texts

# Introduction: Foreword

**Published:** 2010-03-10

**Authors:** Göran Magnusson

**Affiliations:** Dean, Faculty of Medicine

The Department of Neuroscience is a fine example of a successful merger of smaller units. Although the primary reason for the formation of the present Department was managerial, the foresight of the founding members led to both scientific and administrative gains. The international trend towards large university departments is debated. Although it is generally accepted in life science that achievement relies on individual excellence, collaboration and technical resources are becoming increasingly important for competitive research. A university department used to correspond to a discipline. Although many have affection for the traditional disciplines, modern research is not confined to the old domains. Hence, the traditional departments lost much of their identity. Research and education in neuroscience was done in several of the old disciplinary departments, leading to valuable interactions with non-neuroscientists, but many groups became insular with most of their interactions outside the department. Moreover, nobody had the overall responsibility for teaching in neuroscience.

The Department of Neuroscience at the Faculty of Medicine unites clinical and preclinical research and education. Although everyday life of a scientist and teacher does not include exchange of ideas and material with all branches of the department, the focus of a large organization on neuroscience has made a change for this new discipline in Uppsala. It facilitates personal contacts, and arrangement of departmental seminars and symposia with a broad perspective on a scientific problem is an important impetus for novel ideas and collaborative projects.

Progress in the understanding of neuroscience is rapid. A long period of descriptive research laid the foundation for studies on the function of the nervous system. Armed with new methods, neuroscientists are now advancing the understanding of their subject at a rapid pace, the Department of Neuroscience providing support and structure for both research and teaching.
